# Testing for a Debt‐Threshold Effect on Output Growth†

**DOI:** 10.1111/1475-5890.12134

**Published:** 2017-08-30

**Authors:** Sokbae Lee, Hyunmin Park, Myung Hwan Seo, Youngki Shin

**Affiliations:** ^1^ Department of Economics Columbia University Centre for Microdata Methods and Practice Institute for Fiscal Studies; ^2^ Department of Economics University of Chicago; ^3^ Department of Economics Seoul National University; ^4^ Department of Economics McMaster University Economics Discipline Group University of Technology Sydney

**Keywords:** Fiscal policy, government debt, growth, median regression, testing, E6, F34, H60

## Abstract

Using the Reinhart–Rogoff dataset, we find a debt threshold not around 90 per cent but around 30 per cent, above which the median real gross domestic product (GDP) growth falls abruptly. Our work is the first to formally test for threshold effects in the relationship between public debt and median real GDP growth. The null hypothesis of no threshold effect is rejected at the 5 per cent significance level for most cases. While we find no evidence of a threshold around 90 per cent, our findings from the post‐war sample suggest that the debt threshold for economic growth may exist around a relatively small debt‐to‐GDP ratio of 30 per cent. Furthermore, countries with debt‐to‐GDP ratios above 30 per cent have GDP growth that is 1 percentage point lower at the median.

## Policy points

- We find evidence supporting a debt‐threshold effect around 30 per cent on output growth using post‐war cross‐country data.
- More evidence is needed to establish causality between debt threshold and growth.

## I. Introduction

The effect of public debt on economic growth has been an important issue in the recent policy debate on fiscal policies and in academia alike, particularly after the recent financial crisis. Reinhart and Rogoff (2010) successfully brought this issue to a wide audience and many papers followed up the issue. One of the central questions in this debate is that the effect of public debt measured by the debt‐to‐GDP ratio can be heterogeneous for various reasons. For instance, the effect could be different between the short run and the long run. Higher public debt could stimulate the aggregate demand in the short run, which might in turn crowd out private spending in the long run, resulting in reduced output.^1^ Another possibility is that the level of debt may have a highly nonlinear effect on growth.^2^ Furthermore, the literature on public debt emphasises the issue of sustainability – for example, ‘debt overhang’ in Krugman (1988) and ‘fiscal fatigue’ in Ghosh et al. (2013).

We note that the effect of public debt on growth can be heterogeneous in terms of both magnitude and nonlinearity, which leads us to consider a median regression approach to complement the existing literature that is based on mean regression models. In this paper, we test whether there is a threshold effect in the relationship between government debt‐to‐GDP ratio and median real GDP growth rate in advanced economies by applying a recently developed econometric technique to the Reinhart–Rogoff (RR hereafter) dataset.

Our paper is primarily motivated by Reinhart and Rogoff (2010) whose ‘main result is that whereas the link between growth and debt seems relatively weak at “normal” debt levels, median growth rates for countries with public debt over roughly 90 per cent of GDP are about a 1 percentage point lower than otherwise; average (mean) growth rates are several per cent lower’. Herndon, Ash and Pollin (2014) pointed out their spreadsheet errors and claimed that ‘overall evidence refutes RR's claim that public debt*/*GDP ratios above 90 per cent consistently reduce a country's GDP growth’. In the response to their critics, Reinhart and Rogoff (2013bb) stressed among other things that their paper ‘gave significant weight to the median estimates’ because these are less influenced by outliers.

A substantial body of literature since then has been devoted to testing for the threshold effect in the link between public debt and GDP growth but a general consensus has not been reached. Kumar and Woo (2010), Cecchetti, Mohanty and Zampolli (2011), Checherita‐Westphal and Rother (2012) and Baum, Checherita‐Westphal and Rother (2013) obtained evidence supporting the proposed 90 per cent debt threshold. Minea and Parent (2012) estimated a higher debt threshold, around 115 per cent of GDP. However, Caner, Grennes and Koehler‐Geib (2010) and Elmeskov and Sutherland (2012) found the threshold to be around 70 per cent. Hansen (2017) detected the regression kink around 40 per cent. Baglan and Yoldas (2013) and Égert (2015) suggested that the threshold may be even lower, around 20 per cent. For a more comprehensive literature review, refer to Panizza and Presbitero (2013) and Eberhardt and Presbitero (2015). However, the aforementioned papers in the literature did not estimate or test for the threshold effect in terms of the *median* GDP growth rate, although Reinhart and Rogoff emphasised their median estimates. To the best of our knowledge, our paper is the first to focus on the *median* real GDP growth.

In this paper, we contribute to the Reinhart and Rogoff debate by formally testing for a threshold effect in the relationship between public debt and median real GDP growth. The goal of this paper is to examine whether the empirical findings of Reinhart and Rogoff (2010) can be viewed as statistically significant evidence for the existence of the threshold effect around 90 per cent of the debt‐to‐GDP ratio. Although the debate broadly encompasses the link between debt and growth in all economies, we restrict our attention to threshold effects in advanced economies in this paper using the updated RR dataset.^3^ We have found that there is a threshold effect around 30 per cent of the debt‐to‐GDP ratio. Furthermore, countries with debt‐to‐GDP ratios above 30 per cent have GDP growth that is 1 percentage point lower at the median.

The remainder of the paper is organised as follows. In Section II., we describe the set‐up and the methodology. In Section III., we explain the data and give estimation results of median regression. In Section IV., we present the main testing results, and in Section V., we provide the results of the robustness check. We conclude in Section VI.. In the Online Appendix, we describe the construction of the sample used in our empirical work and we provide details of the testing method.

## II. The set‐up and methodology

We use the updated RR dataset^4^ and apply the test for the threshold effect developed in Lee, Seo and Shin (2011). This method allows us to test for the threshold effect in the median regression model when the threshold value is unknown.

### 1. Model specification

Let $y_{c,t}$ be the real GDP of country *c* for year *t*. Let $G_{c,t-1,t}$ be the real GDP growth of the country between the years *t* and t−1, that is, $100\times \left(y_{c,t}-y_{c,t-1}\right)/y_{c,t-1}$. Similarly, let $G_{c,t,t+5}$ be its five‐year forward average growth rate (annualised five‐year growth hereafter) defined as $\left(1/5\right)\sum_{s=0}^{4}G_{c,t+s,t+s+1}$. The annualised five‐year growth is defined so that the period included does not overlap with the period for annual growth. Let ${\mathrm{debt}}_{c,t}$ denote the debt‐to‐GDP ratio of country *c* in year *t* expressed in percentage.

Our main interest is how the debt‐to‐GDP ratio of a country affects its annual and annualised five‐year growth rates. Specifically, we wish to test whether there is a debt‐to‐GDP ratio threshold after which the median growth rates change abruptly. The conditional median function is specified as

(1) $$\begin{array}{ccc} \mathrm{Median}(g_{c,t}|{\mathrm{debt}}_{c,t}) & = & \beta_{1}+\beta_{2}{\mathrm{debt}}_{c,t}\\ & & +\;\left[\alpha_{1}+\alpha_{2}{\mathrm{debt}}_{c,t}\right]\times I\left({\mathrm{debt}}_{c,t}>\gamma \right), \end{array}$$

where $g_{c,t}$ can be either $G_{c,t-1,t}$ or $G_{c,t,t+5}$, I(·) is an indicator function and $\alpha_{1},\;\alpha_{2},\;\beta_{1},\;\beta_{2}\;$and γ are unknown true parameter values that belong to $A_{1},A_{2},B_{1},B_{2}$ and Γ, respectively, which are subsets of the real line, R.^5^ Note that two outcome variables $G_{c,t-1,t}$ and $G_{c,t,t+5}$ capture possibly different effects of debt on growth because the economic channels through which debt affects *contemporary* growth could be different from the channels for *future* growth.

We consider two specifications for the regression model. For the ‘intercept‐only’ model, we consider a conditional median function where only the intercept is allowed to change at the threshold value by imposing *α*_2_ = 0. For the ‘intercept‐and‐slope’ model, changes in both the slope and the intercept are allowed at the threshold value. It follows from equation (1) that the partial effect of debt on growth is given by

(2) $$\frac{\partial \mathrm{Median}(g_{c,t}|{\mathrm{debt}}_{c,t})}{\partial {\mathrm{debt}}_{c,t}}=\beta_{2}+\alpha_{2}\times I\left({\mathrm{debt}}_{c,t}>\gamma \right).$$

Thus, in the intercept‐only model, the partial effect of debt is constant, regardless of the existence of the threshold effect, whereas, in the intercept‐and‐slope model, if the threshold effect exists, the partial effect is β_2_ if the level of debt is below the threshold but $\beta_{2}+\alpha_{2}$ if it is above the threshold. In other words, α_2_ represents the change in the slope coefficient below and above the threshold.

Let $\alpha =[\alpha_{1},\alpha_{2}]$. The null and alternative hypotheses in our setting are

(3) H0:α=0 for  any γ∈Γ versus H1:α≠0 for  some γ∈Γ.

When α=0, there is no threshold effect due to the debt‐to‐GDP ratio, whereas, if α≠0, there exists a threshold effect. The implication of rejecting the null hypothesis *H*_0_ is different between the two specifications. In the intercept‐only model, rejecting *H*_0_ means that there is a jump in the intercept term only, while, in the intercept‐and‐slope model, it implies that there is a change either in the intercept or in the slope (or both).

### 2. Informal description of the testing procedure

There are several testing procedures available in the literature: a sup‐likelihood‐ratio‐type test of Lee, Seo and Shin (2011), a sup‐Wald‐type test of Galvao et al. (2014) and a sup‐score‐type test of Zhang, Wang and Zhu (2014). In this paper, we use the sup‐likelihood‐ratio‐type test of Lee, Seo and Shin (2011) as, in many cases, likelihood ratio tests are known to have desirable properties.

In our analysis, we pool observations as if observations were independent and identically distributed over *c* and *t*. In what follows, we use the subscript *i* for each country–year observation. This is a convenient assumption to start with, but it is also expected that the asymptotic null distribution of the sup‐likelihood‐ratio test statistic is the same for stationary weakly dependent processes, as is the case with the sup‐Wald‐type test of Galvao et al. (2014).^6^

To give an informal description of our testing procedure, we start with the following objective function for the median regression:

$$\begin{array}{ccc} Q_{n}\left(\alpha ,\beta ,\gamma \right): & = & -\frac{1}{n}\sum_{i=1}^{n}\\ & & \times \left|g_{i}\right.\left.-\left\{\beta_{1}+\beta_{2}{\mathrm{debt}}_{i}+\left[\alpha_{1}+\alpha_{2}{\mathrm{debt}}_{i}\right]\times I\left({\mathrm{debt}}_{i}>\gamma \right)\right\}\right|. \end{array}$$

For a given γ∈Γ, define $\hat{\alpha }\left(\gamma \right)$ and $\hat{\beta }\left(\gamma \right)$ to be the estimators that maximise the objective function $Q_{n}\left(\alpha ,\beta ,\gamma \right)$. Let  γ^:= argma xγ∈ΓQn(α^(γ),β^(γ),γ) and

$${\hat{Q}}_{n}:=Q_{n}\left(\hat{\alpha }\left(\hat{\gamma }\right),\hat{\beta }\left(\hat{\gamma }\right),\hat{\gamma }\right).$$

In addition, noting that $Q_{n}\left(\alpha ,\beta ,\gamma \right)$ does not depend on γ when α=0, let

β^:= argma xβ:α=0Qn(α,β,γ) and Q∼n:=Qn(0,β∼,γ).

Define the quasi‐likelihood ratio statistic by

(4) $$QLR_{n}:=n\left({\hat{Q}}_{n}-{\tilde{Q}}_{n}\right).$$

In other words, our test statistic is based on the distance between maximised restricted and unrestricted objective function values. Note that the test statistic defined in equation (4) can also be written as

QLRn= su pγ∈Γn[Qn(α^(γ),β^(γ),γ)−Q∼n].

Thus, the statistic $QLR_{n}$ can be viewed as a sup‐likelihood‐ratio‐type statistic. We can simulate valid *p*‐values of the quasi‐likelihood ratio test, following Lee, Seo and Shin (2011). To implement the test, it is necessary to specify the range of the parameter space Γ of the threshold parameter γ. In all empirical results presented below, we set Γ to be an interval between 10 and 120 per cent of GDP, to include the range of debt thresholds estimated in the previous literature. See the Online Appendix for a detailed description of how to obtain the *p*‐value.

## III. Data and median regression results

The dataset comes from Reinhart and Rogoff (2013a).^7^ This is a revised and corrected version of the data used in Reinhart and Rogoff (2010). For the main analysis, we use the post‐war sample of ‘advanced economies’. The sample covers the years 1946–2009 and includes the following countries: Australia, Austria, Belgium, Canada, Denmark, Finland, France, Germany, Greece, Ireland, Italy, Japan, the Netherlands, New Zealand, Norway, Portugal, Spain, Sweden, the United Kingdom and the United States. The specific country–years included in the sample are described in the Online Appendix.

For the GDP growth of New Zealand, Reinhart and Rogoff (2013a) constructed two different sets of data: first, from Angus Maddison's Database and, then, from the New Zealand Historical Statistics records. We only report the results obtained using the New Zealand Historical Statistics data. We have also conducted the same tests with the Maddison data and the differences in the results are minor.

Before moving to the test results, we present the predicted values from median regression of growth on dummy variables that represent debt‐to‐GDP ratio categories. We use the same debt categories as Reinhart and Rogoff (2010). However, we assign an equal weight to every country–year observation for the whole sample, while they assign an equal weight to every country within a debt category (i.e., the equal weight within each subsample defined by the debt level). In our test for threshold effects, because we test for the existence of any threshold rather than a particular threshold, we do not have ex‐ante debt categories needed to construct the weights used in Reinhart and Rogoff (2010). Hence, for consistency, we use country–year equal weights in estimation of median regression.

**Figure 1:**
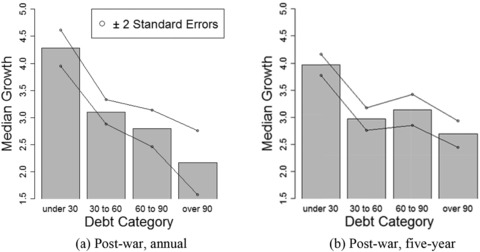
Median regression of growth on debt categories

Figure 1 depicts the predicted median growth and the region within two standard deviations from the predicted median growth. We can observe that the difference in predicted median growth between the categories ‘under 30’ and ‘30 to 60’ is larger than the difference between the categories ‘60 to 90’ and ‘over 90’ for both panels.

## IV. Test results for threshold effects

Table 1 describes the result of the tests for the threshold effect in median annual growth regression and the estimation results for both the restrictive and unrestrictive models. In panel A, the null hypothesis of no threshold effect is rejected in both specifications at the 5 per cent significance level. The estimated threshold is 28 per cent.

**Table 1:** Test for threshold effect in median annual real GDP growth function

	*Intercept‐only*	*Intercept‐and‐slope*
A. Test result		
*n*	1,184	1,184
*p*‐value	0.006	0.026
$\hat{\gamma }$	28	28
B. Restricted model		
${\tilde{\beta }}_{1}$	4.331 (0.148)	4.331 (0.148)
${\tilde{\beta }}_{2}$	−0.022 (0.002)	−0.022 (0.002)
C. Unrestricted model		
${\hat{\beta }}_{1}$	4.540 (0.178)	4.275 (0.459)
${\hat{\beta }}_{2}$	−0.009 (0.004)	0.005 (0.025)
${\hat{\alpha }}_{1}$	−1.074 (0.242)	−0.809 (0.516)
${\hat{\alpha }}_{2}$		−0.015 (0.026)


*Note*: For the ‘intercept‐only’ model, we consider a conditional median function where only the intercept is allowed to change at the threshold value by imposing $\alpha_{2}=0$. For the ‘intercept‐and‐slope’ model, changes in both the slope and the intercept are allowed at the threshold value. ${\tilde{\beta }}_{1}$ and ${\tilde{\beta }}_{2}$ refer to the estimated coefficients for $\beta =(\beta_{1},\beta_{2})$ when the restriction α=0 is imposed. The standard errors are given in parentheses.

In panel B, we report estimation results for the restricted model where α=0 is imposed. The restricted model is simply a standard linear median regression model. The standard errors are given in parentheses. In this model, a 10 percentage point increase in the debt is associated with a 0.22 percentage point decrease in median GDP growth.

In panel C, we estimate the unrestricted model with the estimated threshold parameter $\left(\hat{\gamma }=28\right)$.^8^ In the unrestricted model, the estimated drop in growth at the threshold debt level (28 per cent) is 1 percentage point in the intercept‐only model and 0.8 percentage point in the intercept‐and‐slope model. For the latter model, the estimated β_2_ and α_2_ are 0.005 and –0.015, respectively. However, both coefficients are insignificant at any conventional level.

If we look at country–year observations, countries can be grouped roughly into three groups. One group consists of Belgium, Canada, the UK and the US. For these countries, except for Canada, no observation has a debt level below 28 per cent in the study period.^9^ The second group is composed of Australia, Ireland, New Zealand and Norway. They experienced most years in the 2000s under the debt threshold. The third group is made up of residual countries that enjoyed a substantial period of low debt but moved to the regime above the threshold and stayed there at the end of study period.

Table 2 describes the result of the tests for threshold effect in median annualised five‐year growth regression. The null hypothesis of no threshold effect is rejected in both specifications at any conventional level. The estimated threshold is 32 per cent in the intercept‐only model and 18 per cent in the intercept‐and‐slope model. In the restricted model where α=0 is imposed, a 10 percentage point increase in debt is associated with a 0.12 percentage point decrease in median GDP growth. The estimated coefficients suggest that the negative impact of debt on growth is smaller in future growth compared to contemporaneous growth.

**Table 2:** Test for threshold effect in median five‐year forward average real DGP growth function

	*Intercept‐only*	*Intercept‐and‐slope*
A. Test result		
*n*	1,085	1,085
*p*‐value	0.000	0.001
$\hat{\gamma }$	32	18
B. Restricted model		
${\tilde{\beta }}_{1}$	3.866 (0.103)	3.866 (0.103)
${\tilde{\beta }}_{2}$	−0.012 (0.002)	−0.012 (0.002)
C. Unrestricted model		
${\hat{\beta }}_{1}$	3.992 (0.091)	2.101 (0.363)
${\hat{\beta }}_{2}$	−0.003 (0.001)	0.178 (0.033)
${\hat{\alpha }}_{1}$	−0.909 (0.132)	−1.374 (0.376)
${\hat{\alpha }}_{2}$		−0.184 (0.033)


*Note*: The note from Table 1 applies.

In the unrestricted intercept‐only model, the estimated drop in growth at the threshold debt level is 0.9 percentage point, which is similar to the results in Table 1. For the unrestricted intercept‐and‐slope model, the estimated regression line below the 18 per cent threshold is $2.101+0.178\times {\mathrm{debt}}_{c,t}$ and that above the threshold is $3.475-0.006\times {\mathrm{debt}}_{c,t}$. One can interpret that the 18 per cent threshold is a future‐growth‐maximising optimal debt ratio threshold. However, we need to be careful in drawing this conclusion as our estimation results are not causal but merely descriptive.

## V. Robustness of results

In this section, we check the robustness of our results in three different ways: (i) by searching for the second threshold effect in the subsamples divided by the initial threshold estimate; (ii) by omitting one or more countries from the original sample; (iii) by adding the lagged dependent variable as an additional covariate.

First, we examine whether there is a second threshold below or above the threshold estimated in the previous section. One might bring out the possibility of multiple thresholds in the link between debt and growth, and might justly question whether there is really no evidence of a 90 per cent debt threshold. It is possible that there is another threshold around 90 per cent in samples where the estimated threshold is around 30 per cent, but the effect is not strong enough to be detected. There are fewer observations with debt around 90 per cent than those with debt around 30 per cent in the sample. Thus, it might be more difficult to detect a threshold around 90 per cent compared to around 30 per cent.

**Figure 2:**
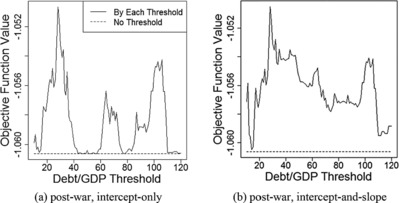
$Q_{n}\left(\hat{\alpha }\left(\gamma \right),\hat{\beta }\left(\gamma \right),\gamma \right)$ for annual growth *Note*: Each panel of the figure plots the profiled value $Q_{n}\left(\hat{\alpha }\left(\gamma \right),\hat{\beta }\left(\gamma \right),\gamma \right)$ of the objective function for each fixed value of γ.

**Figure 3:**
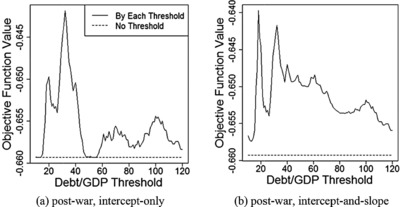
$Q_{n}\left(\hat{\alpha }\left(\gamma \right),\hat{\beta }\left(\gamma \right),\gamma \right)$ for five‐year growth

To examine this issue, we plot the profiled values $Q_{n}\left(\hat{\alpha }\left(\gamma \right),\hat{\beta }\left(\gamma \right),\gamma \right)$ of the objective function for each fixed value of γ in Figures 2 and 3. In Figure 2, there seems to be a second peak near the upper end point of the parameter space; however, with relatively few observations above 100 per cent, it is difficult to conclude whether a threshold really exists there. Likewise, in Figure 3, evidence supporting the presence of another threshold around 90 per cent is weak.

To complement the eyeball examination of figures, in Tables 3 and 4, we report the test results using two subsamples constructed by including only the observations with debt‐to‐GDP ratios below or above the estimated first threshold. Because the possibility of the second peak is more pronounced for annual growth (see Figures 2 and 3), we only present test results using annual growth as the dependent variable. Table 3 reports test results for the intercept‐only model and Table 4 shows those for the intercept‐and‐slope model.

**Table 3:** Test for second threshold effect in median annual real GDP growth function: debt‐to‐GDP below or above the first threshold, ‘intercept‐only’

	*Below 28 per cent*	*Above 28 per cent*
A. Test result		
*n*	415	769
*p*‐value	0.693	0.249
$\hat{\gamma }$	26	77
B. Restricted model		
${\tilde{\beta }}_{1}$	4.275 (0.508)	3.466 (0.236)
${\tilde{\beta }}_{2}$	0.005 (0.028)	−0.009 (0.004)


**Table 4:** Test for second threshold effect in median annual real GDP growth function: debt‐to‐GDP below or above the first threshold, ‘intercept‐and‐slope’

	*Below 28 per cent*	*Above 28 per cent*
A. Test result		
*n*	415	769
*p*‐value	0.499	0.612
$\hat{\gamma }$	11	77
B. Restricted model		
${\tilde{\beta }}_{1}$	4.275 (0.508)	3.466 (0.236)
${\tilde{\beta }}_{2}$	0.005 (0.028)	−0.009 (0.004)


We can observe that the null hypothesis of no threshold effect is not rejected at the 5 per cent significance level for any case. For example, if we look at the subsample above the 28 per cent threshold, there are 769 observations and the estimated threshold parameter is 77 per cent in both intercept‐only and intercept‐and‐slope specifications. However, in either specification, the *p*‐value is not small enough to provide any significant test result. This suggests that there could be a second threshold around 77 per cent but we fail to find any statistically significant evidence. Because the evidence supporting the second threshold effect is weak, we only report estimation results for the restricted model in Tables 3 and 4. Note that the slope coefficient for the subsample consisting of observations below the 28 per cent threshold is modestly positive but insignificant, whereas the slope coefficient above the threshold is –0.009 and significant at the 5 per cent level.

Second, we check whether the results depend on the inclusion/exclusion of any particular country by carrying out the tests with country‐wise subsamples. To that end, we first generate subsamples by omitting one country each time from the original sample. We next generate subsamples by splitting the countries into two groups according to alphabetical order (10 countries in each group) or region (16 European countries and four non‐European countries). Tables 5 and 6 describe the results. We can confirm that the results are scarcely affected by one‐country omissions. When the countries are split into two groups, the estimated threshold points are similar, but the results are only significant for the group of European countries.

**Table 5:** Tests for threshold effects in median annual GDP growth functions, ‘intercept‐only’

	*p*‐*value*	$\hat{\gamma }$	${\tilde{\beta }}_{1}$	${\tilde{\beta }}_{2}$	${\hat{\beta }}_{1}$	${\hat{\beta }}_{2}$	${\hat{\alpha }}_{1}$
Country omitted							
Australia	0.001	28	4.372	−0.023	4.735	−0.010	−1.291
Austria	0.038	28	4.313	−0.021	4.454	−0.010	−0.950
Belgium	0.004	28	4.341	−0.023	4.538	−0.009	−1.117
Canada	0.002	28	4.366	−0.023	4.549	−0.010	−1.134
Denmark	0.020	28	4.395	−0.022	4.587	−0.011	−0.991
Finland	0.005	28	4.396	−0.023	4.600	−0.010	−1.106
France	0.048	28	4.284	−0.021	4.380	−0.009	−0.907
Germany	0.003	28	4.396	−0.022	4.607	−0.010	−1.123
Greece	0.035	28	4.401	−0.024	4.574	−0.013	−0.899
Ireland	0.020	28	4.342	−0.023	4.518	−0.010	−1.039
Italy	0.006	28	4.244	−0.019	4.367	−0.007	−1.006
Japan	0.006	28	4.250	−0.021	4.299	−0.004	−1.146
The Netherlands	0.006	28	4.353	−0.022	4.555	−0.009	−1.095
New Zealand	0.009	28	4.353	−0.022	4.557	−0.010	−1.073
Norway	0.000	29	4.395	−0.023	4.595	−0.009	−1.199
Portugal	0.025	28	4.279	−0.021	4.442	−0.010	−0.958
Spain	0.010	28	4.326	−0.022	4.504	−0.009	−1.083
Sweden	0.011	28	4.353	−0.022	4.541	−0.010	−1.057
United Kingdom	0.064	28	4.409	−0.023	4.562	−0.012	−0.927
United States	0.003	28	4.326	−0.022	4.536	−0.009	−1.145
Countries included							
Australia–Ireland	0.522	24	4.023	−0.013	4.098	0.004	−1.322
Italy–United States	0.036	17	4.620	−0.029	6.352	−0.026	−1.951
European	0.001	28	4.267	−0.021	4.300	−0.001	−1.388
Non‐European	0.012	13	5.316	−0.036	8.492	−0.016	−4.513


**Table 6:** Tests for threshold effects in median annual GDP growth functions, ‘intercept‐and‐slope’

	*p*‐*value*	$\hat{\gamma }$	${\tilde{\beta }}_{1}$	${\tilde{\beta }}_{2}$	${\tilde{\beta }}_{1}$	${\tilde{\beta }}_{2}$	$\;\;{\hat{\alpha }}_{1}$	$\;\;\;{\hat{\alpha }}_{2}$
Country omitted								
Australia	0.005	28	4.372	−0.023	4.844	−0.017	−1.428	0.008
Austria	0.105	28	4.313	−0.021	4.172	0.009	−0.587	−0.020
Belgium	0.015	28	4.341	−0.023	4.275	0.005	−0.855	−0.014
Canada	0.016	28	4.366	−0.023	4.222	0.008	−0.807	−0.018
Denmark	0.071	28	4.395	−0.022	4.526	−0.008	−0.910	−0.004
Finland	0.024	28	4.396	−0.023	4.489	−0.004	−0.996	−0.006
France	0.141	28	4.284	−0.021	4.264	−0.002	−0.791	−0.008
Germany	0.017	28	4.396	−0.022	4.040	0.021	−0.480	−0.032
Greece	0.109	28	4.401	−0.024	4.248	0.006	−0.573	−0.019
Ireland	0.068	28	4.342	−0.023	4.594	−0.015	−1.115	0.005
Italy	0.047	28	4.244	−0.019	4.633	−0.019	−1.298	0.013
Japan	0.010	28	4.250	−0.021	3.885	0.025	−0.596	−0.031
The Netherlands	0.020	28	4.353	−0.022	4.286	0.005	−0.826	−0.014
New Zealand	0.029	28	4.353	−0.022	4.286	0.005	−0.767	−0.015
Norway	0.005	28	4.395	−0.023	4.366	0.006	−0.946	−0.015
Portugal	0.084	28	4.279	−0.021	4.212	0.006	−0.709	−0.016
Spain	0.041	28	4.326	−0.022	4.655	−0.019	−1.234	0.010
Sweden	0.036	28	4.353	−0.022	4.248	0.006	−0.729	−0.016
United Kingdom	0.332	28	4.409	−0.023	4.275	0.005	−0.616	−0.017
United States	0.009	28	4.326	−0.022	4.275	0.005	−0.884	−0.014
Countries included								
Australia–Ireland	1.000	24	4.023	−0.013	3.960	0.018	−1.155	−0.015
Italy–United States	0.218	13	4.620	−0.029	11.018	−0.700	−6.444	0.673
European	0.002	28	4.267	−0.021	3.709	0.035	−0.551	−0.040
Non‐European	0.135	13	5.316	−0.036	9.899	−0.145	−5.920	0.130


To check for the possibility of a second threshold among European countries, we repeated the same exercises as in Tables 3 and 4 for European countries only. The corresponding test results are given in Table 7. It can be seen that the empirical results in Tables 3 and 4 are more or less replicated in Table 7.

**Table 7:** Test for second threshold effect in median annual real GDP growth function: debt‐to‐GDP below or above the first threshold, using only European countries

	Intercept‐only		Intercept‐and‐slope	
	*Below 28*	*Above 28*	*Below 28*	*Above 28*
A. Test result				
*n*	383	562	383	562
*p*‐value	0.759	0.156	0.773	0.449
$\hat{\gamma }$	17	77	11	77
B. Restricted model				
${\tilde{\beta }}_{1}$	3.709 (0.535)	3.157 (0.271)	3.709 (0.535)	3.157 (0.271)
${\tilde{\beta }}_{2}$	0.035 (0.029)	−0.006 (0.005)	0.035 (0.029)	−0.006 (0.005)

Finally, we consider further tests by adding the lagged dependent variable as an explanatory variable when the dependent variable is annual growth. We consider the following three specifications:

(5) $$\begin{array}{ccc} & & \text{Median}(G_{c,\;t-1,\;t}|{\mathrm{debt}}_{c,\;t})\\ & & \;=\;\beta_{1}+\;\beta_{2}{\mathrm{debt}}_{c,\;t}+\;\beta_{3}G_{c,\;t-2,\;t-1}+\;\alpha_{1}\times I\left({\mathrm{debt}}_{c,\;t}>\gamma \right), \end{array}$$

(6) $$\begin{array}{ccc} & & \text{Median}(G_{c,\;t-1,\;t}|{\mathrm{debt}}_{c,\;t})\\ & & \;=\;\beta_{1}+\;\beta_{2}{\mathrm{debt}}_{c,\;t}+\;\beta_{3}G_{c,\;t-2,\;t-1}+\;\left[\alpha_{1}+\alpha_{2}{\mathrm{debt}}_{c,\;t}\right]\\ & & \;\times I({\mathrm{debt}}_{c,\;t}>\gamma ), \end{array}$$

(7) $$\begin{array}{ccc} & & \text{Median}(G_{c,\;t-1,\;t}|{\mathrm{debt}}_{c,\;t})\\ & & \;=\;\beta_{1}+\;\beta_{2}{\mathrm{debt}}_{c,\;t}+\;\beta_{3}G_{c,\;t-2,\;t-1}+\;[\alpha_{1}+\alpha_{2}{\mathrm{debt}}_{c,\;t}\\ & & \;+\;\alpha_{3}G_{c,\;t-2,\;t-1}]\times I\left({\mathrm{debt}}_{c,\;t}>\gamma \right). \end{array}$$

Table 8 indicates that the threshold effect seems to disappear altogether. The test fails to reject the null hypothesis in all cases at the 5 per cent significance level. The coefficient for the lagged dependent variable is around 0.5 and highly significant. The estimation results imply that a 10 percentage point increase in the debt is associated with 0.08 percentage point decrease in annual growth. This result further casts doubt on the existence of a threshold effect around 90 per cent of the debt‐to‐GDP ratio. In summary, we do not find any credible evidence to support the existence of a threshold around 90 per cent in median regression.

**Table 8:** Test for threshold effect in median annual real GDP growth function with lagged dependent variable

	*Model 5*	*Model 6*	*Model 7*
A. Test result			
*n*	1,161	1,161	1,161
*p*‐value	0.105	0.220	0.220
$\hat{\gamma }$	28	28	77
B. Restricted model			
${\tilde{\beta }}_{1}$	2.104 (0.161)	2.104 (0.161)	2.104 (0.161)
${\tilde{\beta }}_{2}$	−0.008 (0.002)	−0.008 (0.002)	−0.008 (0.002)
${\tilde{\beta }}_{3}$	0.475 (0.025)	0.475 (0.025)	0.475 (0.025)


*Note*: ${\tilde{\beta }}_{3}\;$is the coefficient that corresponds to the lagged dependent variable.

## VI. Conclusion

After testing for threshold effects in the link between public debt‐to‐GDP ratio and median growth, we find no evidence of the threshold effect at 90 per cent of the debt‐to‐GDP ratio that is generally applicable to all countries. Instead, our findings suggest that a debt threshold, if it exists, may be around 30 per cent of GDP. However, more evidence is needed to establish any credible link between such debt threshold and growth.

Our paper has some limitations. Although we carry out formal hypothesis testing for the existence of a debt threshold, our work is simply descriptive by its nature. As a result, we do not consider the issue of causality in the debt–growth relationship. One could perhaps investigate the issue of causality by exploiting panel data with a structural econometric model that is guided by economic theory. In addition, it might be fruitful to allow for country‐specific heterogeneity in the debt–growth relationship. Country‐specific variations in macro‐economic variables such as inflation and interest rates might provide the key to uncover economic channels through which public debt affects growth.^10^ These are important future research topics.

## Supporting information

• Online Appendix

## Footnotes

1
Eberhardt and Presbitero, 2015; Chudik et al., 2017.

2 Mauro et al., 2015.

3 Reinhart and Rogoff (2013a).

4 Reinhart and Rogoff (2013a).

5 Note that the average five‐year growth rate is taken within a country, but the median regression in the paper is concerned with comparison across countries over time. Therefore, it is possible to consider the average five‐year growth rate as the dependent variable in equation (1). Alternatively, one can consider the median five‐year growth rate as the dependent variable. This would correspond to a different median regression model.

6 Lee, Seo and Shin (2011) did not consider dependent observations; however, we expect that the asymptotically valid *p*‐value can be obtained in the same way as described in this paper, in view of the asymptotic equivalence result obtained in Galvao et al. (2014) for the sup‐Wald‐type test.

7 See http://www.carmenreinhart.com/data/.

8 When α≠0, the threshold parameter can be estimated at a rate of *n* − 1 when the sample *n* goes to infinity. This implies that standard errors for the (β, α) coefficients can be obtained as if γ were known.

9 Only two years were below the threshold for Canada (27 per cent in 2007 and 23 per cent in 2008).

10 For example, see Crafts (2016) regarding the UK's experience.
