# Supplementary material for: Testing for a Debt‐Threshold Effect on Output Growth
Source: Fisc Stud. 2017 Aug 30;38(4):701–17. doi: 10.1111/1475-5890.12134 (PMC5726385; doi:10.1111/1475-5890.12134)
Supplement: Supplementary file 1 — • Online Appendix [file FISC-38-701-s001.docx]

**Testing for a Debt-Threshold Effect on Output Growth: Online Appendix**

Sokbae Lee, Hyunmin Park, Myung Hwan Seo and Youngki Shin

## I. Data construction

Following the ‘Guide to Changes’ in Reinhart and Rogoff (2013a), we use the ‘Spain new’ data that include the years 1959–1980 and we conduct each test twice. First, we use ‘New_Zealand_old’ data constructed by Reinhart and Rogoff from Angus Maddison’s database. These have been carried over to the Total Economy Database. Second, we use the ‘New_Zealand_new’ data constructed by Reinhart and Rogoff from the New Zealand Historical Statistics (2003).

Following the comments in the worksheet, we exclude years 1940–1945 for the United Kingdom and 1941–1944 for the United States. In the worksheet, debt-to-GDP ratios in years 2008–2009 for Greece are missing, while the two observations are included in the calculation of mean and median growth rate for the ‘90 or above’ debt-to-GDP ratio category. Hence, we augment the worksheet data with debt-to-GDP ratios of the Reinhart–Rogoff series provided on Carmen Reinhart’s web site[^[[1]](#footnote-1)^](#_bookmark28) to fill in the debt-to-GDP ratios for the two observations (which are 109.748642014544 for 2008 and 126.8 for 2009). In the worksheet, the post-war (1946–2009) summary statistics include only years 1951–2009 for Italy, although both the debt-to-GDP ratio and the real GDP growth rate in 1946 for Italy are available. Hence, we also exclude this observation when constructing the post-war sample in our analysis.

With the exception of years 2008–2009 for Greece, we have deleted all observations that have either the debt-to-GDP ratio or the real GDP growth rate missing. For the post-war sample, 12 observations were deleted (1976–1979 Denmark; 1949, 1973–1977 France; 1951–1952 Portugal). Dropping these observations with missing values allowed us to reproduce the summary statistics reported in ‘Final including New Zealand (NZ Historical Statistics GDP)’ and ‘Final including New Zealand (Maddison GDP)’ in Table 1 of the errata. The final data include 1,184 observations for the post-war sample.

TABLE A.1

Summary statistics of GDP growth by debt category

| *Growth interval* | *Debt category* | *n* | *Mean* | *Std dev.* | *Min* | *Max* |
| --- | --- | --- | --- | --- | --- | --- |
| Annual | less than 30 | 445 | 4.309 | 2.944 | -6.244 | 18.902 |
| Annual | 30 to 60 | 442 | 3.076 | 2.930 | -7.5 | 27.329 |
| Annual | 60 to 90 | 199 | 2.931 | 2.640 | -4.349 | 11.441 |
| Annual | over 90 | 98 | 2.145 | 3.072 | -10.942 | 15.216 |
| Five-year | less than 30 | 421 | 4.045 | 1.912 | -1.449 | 10.522 |
| Five-year | 30 to 60 | 399 | 3.103 | 1.527 | -1.031 | 10.195 |
| Five-year | 60 to 90 | 181 | 3.267 | 1.656 | -0.386 | 9.652 |
| Five-year | over 90 | 84 | 2.751 | 1.065 | 0.047 | 4.880 |

*Note:* There is no observation in any of the samples with debt/GDP that equals exactly 30, 60 or 90 percent.

FIGURE A.1

Box plots of growth by debt category

| 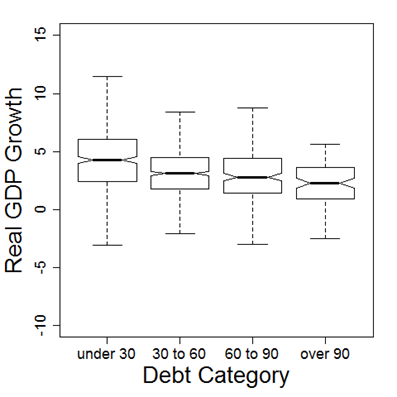 | 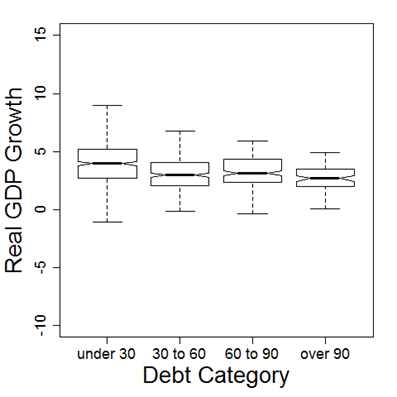 |
| --- | --- |
| (a) post-war, annual | (b) post-war, five-year |

For the five-year forward average real GDP growth, we take an average of annual growth rates included in the five-year forward window. The observation was dropped from the sample if any one of $G_{c, t, t+1}$, $G_{c, t+1, t+2}$, $G_{c, t+2, t+3}$ , $G_{c, t+3, t+4}$ or $G_{c, t+4, t+5}$ was missing. See Table A.1 and Figure A.1 for the summary of the data.

# II. Details of the testing procedure

Here, let $x_{i}=(1, \mathrm{debt}_{i})'$, and $w_{i}=\mathrm{debt}_{i}$. Also, let $z_{i}=1$ for the intercept-only model and $z_{i}=x_{i}$ for the intercept-and-slope model, respectively. [Lee,](#_bookmark47) [Seo and Shin](#_bookmark47) ([2011](#_bookmark47)) showed among other things that, under the null hypothesis ([3](#_bookmark2)), the limiting distribution of $\mathrm{QLR}_{n}$ is the same as the limiting distribution of

$$\mathrm{QLR}_{n}:= \frac{1}{2}\sup_{\gamma}\left[ G_{n}^{j}\left( \gamma\right)^{'}\hat{V}\left( \gamma\right)^{-1}G_{n}^{j}\left( \gamma\right)-\bar{G}_{n}^{j^{'}}\bar{V}^{-1}\bar{G}_{n}^{j} \right]$$

where

$$G_{n}^{j}\left( \gamma\right):= \frac{1}{\sqrt{n}}\sum_{i=1}^{n} \left( x_{i}^{'}, z_{i}^{'}\times I\left( w_{i}>\gamma\right) \right)^{'}[\tau-I(u_{\mathrm{ij}}\leq\tau)],$$

$$\bar{G}_{n}^{j}:= \frac{1}{\sqrt{n}}\sum_{i=1}^{n} x_{i}[\tau-I(u_{\mathrm{ij}}\leq\tau)],$$

$$\hat{V}\left( \gamma\right):= \frac{1}{nh_{n}}\sum_{i=1}^{n} \left( x_{i}^{'}, z_{i}^{'}\times I\left( w_{i}>\gamma\right) \right)^{'}\left( x_{i}^{'}, z_{i}^{'}\times I\left( w_{i}>\gamma\right) \right)\times K\left( \frac{Y_{i}-(x_{i}^{'}, z_{i}^{'}\times I(w_{i}>\gamma)\hat{\beta}}{h_{n}} \right),$$

$$\bar{V}:=\frac{1}{\mathrm{nh}_{n}}\sum_{i=1}^{n} x_{i}x_{i}^{'}K\left( \frac{Y_{i}-x_{i}^{'}\bar{\beta}}{h_{n}} \right).$$

Here, $u_{\mathrm{ij}}$ are iid random variables following the uniform distribution on $[0, 1]$, $h_{n}$ is the bandwidth, and $K$ is a kernel function. Here, the subscript $j$ denotes each simulation draw. We use the standard normal probability density function as the kernel function. We simulate the distribution of $\mathrm{QLR}_{j}$ and calculate the p-value for $\mathrm{QLR}_{n}$ accordingly.

In simulating the p-value, it is necessary to choose the bandwidth $h_{n}$. It was set to $\hat{\sigma}\times n^{{-1}/5}$, where $\hat{\sigma}$ is the sample standard deviation of $\bar{u}_{i, t}=y_{i, t}-x_{i,t}^{'}\bar{\beta}$. We have also tried different bandwidths that are 0.5, 1.5 and 2 times this size. The results are not sensitive to bandwidth selection. The parameter space [10%, 120%] of the threshold is approximated by a grid such that $\Gamma=\left\{ \gamma: \gamma=10+k, k=0, 1, 2, \ldots, 110 \right\}.$ Note that the end points of the parameter space [10%, 120%] are about 7 and 98 percentiles in the sample.

1. <http://www.carmenreinhart.com/data/browse-by-topic/topics/9/>, last updated on November 15, 2010, downloaded on December 24, 2013. [↑](#footnote-ref-1)
